# Preoperative platelet count, preoperative hemoglobin concentration and deep hypothermic circulatory arrest duration are risk factors for acute kidney injury after pulmonary endarterectomy: a retrospective cohort study

**DOI:** 10.1186/s13019-019-1026-4

**Published:** 2019-12-30

**Authors:** Congya Zhang, Guyan Wang, Hui Zhou, Guiyu Lei, Lijing Yang, Zhongrong Fang, Sheng Shi, Jun Li, Zhiyan Han, Yunhu Song, Sheng Liu

**Affiliations:** 1 Department of Anesthesiology, Fuwai Hospital, National Center for Cardiovascular Diseases, Chinese Academy of Medical Sciences, Peking Union Medical College, Beijing, People’s Republic of China; 2 Department of Anesthesiology, Beijing Tongren Hospital, Capital Medical University, Beijing, People’s Republic of China; 3 Department of Anesthesiology, Ruijin Hospital, Shanghai Jiaotong University School of Medicine, Shanghai, People’s Republic of China; 4 Department of Cardiovascular Surgery, Fuwai Hospital, National Center for Cardiovascular Diseases, Chinese Academy of Medical Sciences, Peking Union Medical College, Beijing, People’s Republic of China

**Keywords:** Acute kidney injury, Chronic thromboembolic pulmonary hypertension, Pulmonary endarterectomy, Risk factors

## Abstract

**Background:**

Acute kidney injury (AKI) is a major postoperative morbidity of patients undergoing cardiac surgery and has a negative effect on prognosis. The kidney outcomes after pulmonary endarterectomy (PEA) have not yet been reported; However, several perioperative characteristics of PEA may induce postoperative AKI. The objective of our study was to identify the incidence and risk factors for postoperative AKI and its association with short-term outcomes.

**Methods:**

This was a single-center, retrospective, observational, cohort study. Assessments of AKI diagnosis was executed based on the Kidney Disease Improving Global Outcomes (KDIGO) criteria.

**Results:**

A total of 123 consecutive patients who underwent PEA between 2014 and 2018 were included.

The incidence of postoperative AKI was 45% in the study population. Stage 3 AKI was associated with worse short-term outcomes and 90-day mortality (*p* < 0.001, *p* = 0.002, respectively). The independent predictors of postoperative AKI were the preoperative platelet count (OR 0.992; 95%CI 0.984–0.999; *P* = 0.022), preoperative hemoglobin concentration (OR 0.969; 95%CI 0.946–0.993; *P* = 0.01) and deep hypothermic circulatory arrest (DHCA) time (OR 1.197; 95%CI 1.052–1.362; *P* = 0.006) in the multivariate analysis.

**Conclusion:**

The incidence of postoperative AKI was relatively high after PEA compared with other types of cardiothoracic surgeries. The preoperative platelet count, preoperative hemoglobin concentration and DHCA duration were modifiable predictors of AKI, and patients may benefit from some low-risk, low-cost perioperative measures.

## Introduction

Chronic thromboembolic pulmonary hypertension (CTEPH) is a rare chronic form of pulmonary hypertension (PH) characterized by pathological changes in the pulmonary arteries and the presence of an occlusive tissue thromboembolism in the arterial lumen. CTEPH may lead to a gradual deterioration of PH, followed by right ventricular failure and eventually death [1–4]. The incidence of CTEPH is 3–30 per million in the general population and 0.4 to 9.1% in acute pulmonary embolism survivors [5]. Without appropriate treatment, the mortality rate is 90% after 3 years in patients with a mean pulmonary artery pressure greater than 50 mmHg [6].

Pulmonary endarterectomy (PEA) is recommended as the gold standard therapy by current guidelines [2, 7–10]. In the setting of surgery, a complete bilateral pulmonary thromboendarterectomy with cardiopulmonary bypass (CPB) and deep hypothermic circulatory arrest (DHCA) offers these patients the best chance of improved long-term outcomes. The UC San Diego group, one of the most experienced centers in the world, has reported 5-year and 10-year survival rates of 82 and 75%, respectively, and an in-hospital mortality rate of only 2.2% [11].

Acute kidney injury (AKI) is a common postoperative complication in patients undergoing cardiothoracic surgery and is associated with increased mortality and major adverse outcomes [12–16]. Due to different definitions and types of surgery, the incidence of postoperative AKI is also different, especially higher in thoracic aortic surgery as a result of prolonged procedure time, extended hypothermic circulatory arrest time, and complex technique [13–15, 17, 18].

The PEA procedure has the similar characteristics to thoracic aortic surgery. However, numerous studies have reported on the PEA-related complications [19–21], such as reperfusion lung edema and bleeding. Little is known about the incidence of AKI after PEA, and the association of postoperative AKI and outcome is lacking. In this study, our objective was to assess the incidence of AKI after PEA. We also aimed to explore potential risk factors in patients undergoing PEA and to clarify the relationship between AKI and short-term outcomes.

## Methods

### Study population

This study was approved by the Ethics Committees of the authors’ center. We included consecutive patients with CTEPH undergoing PEA between January 1, 2014 and August 31, 2018 in this retrospective observational study. We excluded patients whose final diagnosis at discharge was something other than CTEPH(*n* = 3) and whose pre- or postoperative serum creatinine (SCr) data were missing (*n* = 1). The final study population was 123 patients.

### Surgical management

The surgical technique has been described in detail before [10, 22, 23] and involved bilateral pulmonary endarterectomy through a median sternotomy approach under CPB with DHCA. During CPB, core cooling to approximately 20 °C and periods of circulatory arrest were limited to 20 min at a time to provide a bloodless field for better recognition of the dissection plane and removal of the thromboembolism.

### Data definitions

A standard set of perioperative data was collected from the Fuwai Hospital electronic medical record system. Two independent investigators checked the quality of the data by performing regular crosschecks.

AKI was determined and classified according to the Kidney Disease Improving Global Outcomes (KDIGO) criteria [24], which are the most widely recognized diagnostic criteria. We defined postoperative AKI as an increase in SCr over 50% of the baseline level during the first 7 days after surgery or an increase in SCr by 0.3 mg/dl within 48 h after surgery. The baseline SCr was defined as the concentration measured closest to the time before surgery (generally within 3 days, or no more than 7 days from surgery). The severity of AKI was graded according to the criteria listed in Table 1. We recorded the 90-day mortality as the primary outcome and the mechanical ventilation time (MVT), length of intensive care unit stay (LOIS) and postoperative length of hospital stay (p-LOHS) as secondary outcomes to assess the association of AKI with short-term outcomes.

**Table 1:** KDIGO Criteria

Stage	Serum Creatinine Increase
1	1.5~1.9 times baseline or ≥ 0.3 mg/dL (26.5umol/L) increase
2	2.0~2.9 times increase
3	≥3.0 times baseline or increase in serum creatinine to ≥4.0 mg/dL (353.6umol/L) or initiation of renal replacement therapy

### Statistical analysis

Continuous variables are presented as the mean (± standard deviation (SD)) for normally distributed variables or the median and interquartile range (IQR) and 25 – 75th percentiles for non-normally distributed variables, and categorical variables are presented as frequencies with percentages. Student’s t-test or the Mann-Whitney U-test were used to compare continuous variables, and the chi-square test or Fisher’s exact test were used to compare categorical variables among different groups. Logistic regression models were used to identify univariate and multivariate risk factors for AKI. All significant univariates were included in the multivariate model. A stepwise backward method was chosen for regression analysis. The Hosmer-Lemeshow goodness-of-fit statistic was performed to evaluate the fitness of the logistic regression model, and receiver operating characteristic (ROC) curve analysis was used to assess its discrimination. For all analyses, a probability value of less than 0.05 was considered statistically significant. Data were analyzed using SPSS 20.0.

## Results

### Study population

**Fig. 1:**
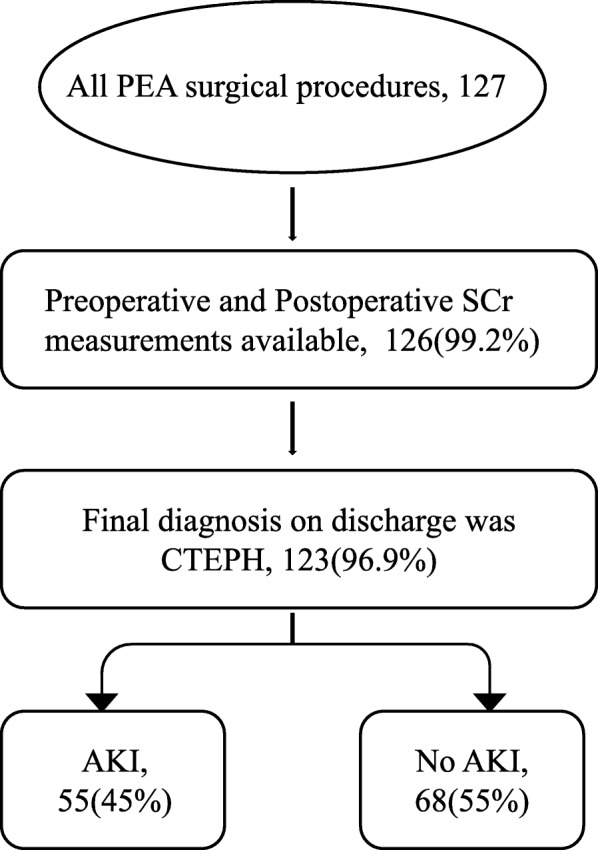
Flow diagram of the patients undergoing PEA surgery

There were 127 CTEPH patients who received PEA at Fuwai Hospital during the last 5 years. We excluded 3 patients with a final diagnosis of something other than CTEPH at discharge and 1 patients with missing preoperative SCr data. Ultimately, 123 patients were included in the study (Fig. 1).

### Patient characteristics

Eighty-two patients (66.7%) were male, with a mean age of 46.5 ± 12.8 years (range, 18-78 years). All patients were diagnosed with CTEPH. Other comorbidities included hypertension (16.2%), diabetes (3.3%), and coronary artery disease (8.9%). Concerning risk factors for CTEPH, 26 (21.1%) patients had acute pulmonary embolism, 49 (39.8%) patients were diagnosed with deep venous embolism, 9 (7.3%) patients were diagnosed with anti-phospholipid syndrome, and 17 (13.8%) patients had a history of acute thrombolysis. All study participants underwent PEA at the authors’ center. The mean CPB time in this study cohort was 243 ± 170 min, and the mean duration of DHCA was 35 ± 17 min.

Concerning preoperative renal function, the SCr levels of 12 (9.8%) patients were greater than 1.2 mg/dL, but none of them had SCr levels greater than 2 mg/dL. There was no difference between the AKI and non-AKI groups in preoperative renal function.

### Postoperative AKI: incidence and risk factors

The overall incidence of postoperative AKI diagnosed according to the KDIGO criteria was 45% (*n* = 54); 37 (31%) patients were in stage 1, 10 (8%) patients were in stage 2, and 7 (6%) patients were in stage 3 AKI. Demographic and perioperative variables of the 4 groups are compared in the Table 2.

**Table 2:** The demographics and perioperative data of study patients(*n* = 123)

Variables	No AKI	Stage			*P* Value^*^
		1	2	3	
Patient population	68	38	10	7	
Demographic data					
Age (y)	45.0 (32.5, 56.0)	52.0 (41.5, 54.0)	55.5(44.0, 64.3)	51.0 (25.0, 60.0)	0.063
Male sex (%)	47(69.1%)	20(52.6%)	8(80%)	7(100%)	0.798
BMI (kg/m^2^)	23.5 (21.1, 26.0)	22.6 (20.0, 25.6)	22.2 (21.0, 23.6)	23.7 (19.6, 25.3)	0.222
Medical history					
Diabetes (%)	3(4.4%)	1(2.6%)	0	0	0.768
Hypertension (%)	13(19.1%)	4(10.5%)	2(20.0%)	1(14.3%)	0.340
CAD (%)	7(10.3%)	2(5.3%)	1(10.0%)	1(14.3%)	0.559
Smoking (%)	16(23.5%)	9(23.7%)	3(30.0%)	3(42.9%)	0.634
Risk factors for CTEPH					
Deep venous embolism (%)	28(41.2%)	16(42.1%)	4(40.0%)	1(14.3%)	0.736
Acute pulmonary embolism (%)	14(20.6%)	9(23.7%)	3(30%)	0	0.868
History of acute thrombolysis (%)	12(17.6%)	4(10.5%)	1(10.0%)	0	0.172
Anti-phospholipid syndrome (%)	6(8.8%)	1(2.6%)	1(10.0%)	1(14.3%)	0.715
Baseline (Preoperative) renal function					
Preoperative serum creatinine (mg/dL)	0.92 (0.82, 1.10)	0.86 (0.76, 1.04)	1.01 (0.87, 1.08)	1.13 (1.01,1.20)	0.584
Renal insufficiency (%)	7(10.3%)	2(5.3%)	1(10.0%)	2(28.6%)	0.823
Preoperative cardiac status					
Anteroposterior diameter of right ventricular (mm)	29.0 (25.5, 37.0)	31.0 (26.5, 39.0)	36.0 (25.0, 46.0)	46.0 (28.0, 58.0)	0.066
Internal diameter of major pulmonary artery (mm)	29.0 (25.0, 31.8)	30.0 (24.5, 34.5)	31.5 (23.5, 37.5)	33.0 (31.0, 45.0)	0.031
Left ventricular ejection fraction (%)	65 (60, 71)	67 (61, 73)	73 (62, 75)	67 (55, 70)	0.255
NYHA class III	45(66.2%)	27(71.1%)	8(80.0%)	6(85.7%)	0.161
NYHA class IV	1(1.5%)	2(5.3%)	0	1(14.3%)	0.467
Right heart catheterization Data					
Mean pulmonary arterial pressure (mmHg)	47.0 (40.0, 60.0)	48.5 (39.8,53.5)	51.0 (47.5, 62.5)	55.0 (46.8, 62.3)	0.909
Pulmonary vascular resistance (Wood U)	8.3 (5.4, 13.4)	7.6 (6.2, 10.1)	10.2 (6.5, 15.4)	6.8 (5.5, 14.1)	0.901
Cardiac output (L min^−1^)	5.0 (3.8, 5.7)	4.5 (3.9, 5.3)	3.4 (3.2, 5.3)	4.5 (3.8, 6.3)	0.125
Laboratory tests					
Preoperative platelet count (10^9^/L)	212 (173, 241)	174 (127, 219)	165 (133, 208)	194 (105, 202)	0.002
Preexisting thrombocytopenia (platelet count< 150,109/L) (%)	11(16.2%)	9(23.7%)	3(30%)	3(42.9%)	0.134
Preoperative hemoglobin (g/L)	149 (137, 157)	139 (113, 151)	153 (127, 158)	155 (138, 163)	0.065
Preexisting anemia (%)	2(2.9%)	8(21.1%)	1(10%)	1(14.3%)	0.005
intraoperative blood product use (%)					
Red blood cells	1(1.5%)	0	0	1(14.3%)	0.008
Fresh frozen plasma	4(5.9%)	2(5.3%)	2(20%)	1(14.3%)	0.052
Platelets	2(2.9%)	4(10.5%)	3(30%)	1(14.3%)	0.007
Surgical details					
Procedure time (min)	325 (296, 346)	332 (301, 364)	373 (313, 384)	383 (335, 468)	0.09
CPB duration (min)	222 (187, 243)	229 (190, 256)	249 (213, 275)	252 (236, 299)	0.045
Aortic cross-clamp time (min)	104 (89, 124)	117 (86, 131)	100 (84, 135)	136 (113, 149)	0.145
DHCA duration (min)	37 (20, 45)	41 (31, 48)	36 (27, 49)	47 (36, 58)	0.014
Lowest rectal temperature (°C)	19.6 (18.9, 20.6)	19.1 (18.7, 20.0)	19.4 (18.4, 19.9)	19.2 (18.7, 20.2)	0.074
Lowest nasopharyngeal temperature (°C)	18.0 (17.7,18.6)	18.1 (17.6,18.5)	18.2 (18.0,19.0)	18.0 (17.7,18.7)	0.555
Outcomes (%)					
RRT	0	0	0	4(57.1%)	0.08
ECMO	1(1.5%)	0	0	2(28.6%)	0.852
Delirium	6(8.8%)	5(13.2%)	2(20.0%)	1(14.3%)	0.109
Reperfusion pulmonary edema	3(4.4%)	0	1(10.0%)	4(57.1%)	0.497
Pulmonary infection	27(39.7%)	18(47.4%)	6(60.0%)	7(100%)	0.028
Re-exploration	3(4.4%)	2(5.3%)	1(10.0%)	0	1.000
Tracheotomy	2(2.9%)	0	3(30%)	4(57.1%)	0.085
Pericardial effusion	4(5.9%)	3(7.9%)	1(10.0%)	1(14.3%)	0.740
Stroke	1(1.5%)	0	0	0	1.000
Debridement	2(2.9%)	0	0	0	0.572

*BMI* Body mass index, *CAD* Coronary artery disease; Renal insufficiency, preoperative serum creatinine > 1.2 mg/dL; anemia, hemoglobin< 120 g/L in male and hemoglobin< 110 g/L in female, *CPB* Cardiopulmonary bypass, *DHCA* Deep hypothermia circulatory arrest, *RRT* Renal replacement therapy, *ECMO* Extracorporeal membrane oxygenation

^*^Comparisons between patients without AKI and with AKI

**Fig. 2:**
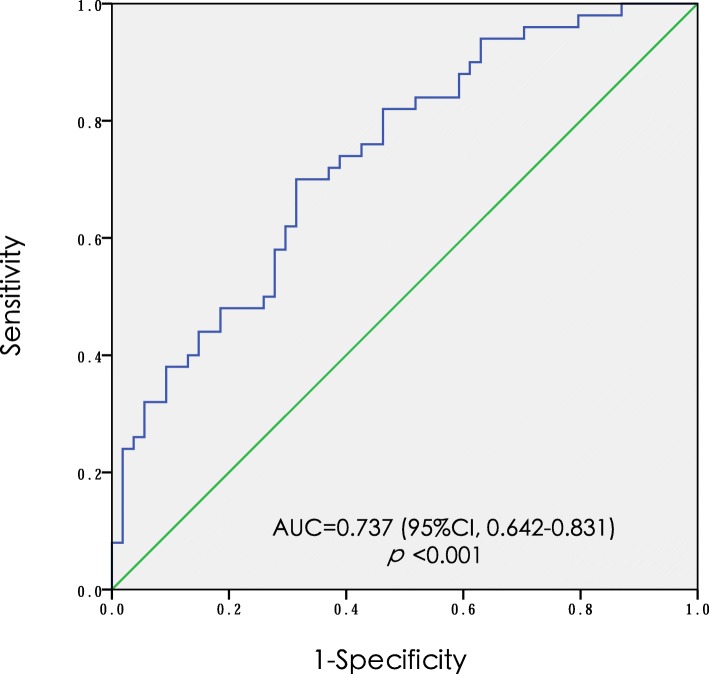
The area under ROC curve for acute kidney injury. * ROC, receiver operating characteristic; AUC, area under curve; CI, confidence interval

The independent predictors of AKI were the preoperative platelet count, preoperative hemoglobin concentration, and DHCA duration. The odds ratios (ORs) and 95% confidence intervals (CIs) for these predictors of AKI are as follows: preoperative platelet count of 0.992 (0.984–0.999); preoperative hemoglobin concentration of 0.969 (0.946–0.993); and DHCA duration of 1.197 (1.052–1.362) (Table 3). The appropriateness of the logistic regression model was authorized by the Hosmer-Lemeshow goodness-of-fit statistic (*p* = 0.505). The area under the curve of ROC for AKI was 0.737 (95%CI, 0.642–0.831) (Fig. 2).

**Table 3:** Univariate and Multivariate Analysis of Risk Factors for Acute Kidney Injury

	Univariate analysis			Multivariate analysis		
	Odds Ratio	95% Confidence Interval	*p* Value	Odds Ratio	95% Confidence Interval	*p* Value
Age	1.029	0.981–1.041	0.052			
Male sex	0.747	0.324–1.721	0.493			
BMI	0.933	0.830–1.049	0.249			
Diabetes	0.347	0.035–3.450	0.666			
Hypertension	0.430	0.149–1.237	0.111			
CAD	0.584	0.160–2.129	0.411			
Smoking	1.352	0.567–3.223	0.496			
Preoperative serum creatinine	0.990	0.968–1.012	0.364			
Renal insufficiency	0.871	0.261–2.914	0.823			
Preoperative platelet count	0.992	0.986–0.998	0.016	0.992	0.984–0.999	0.022
thrombocytopenia	1.943	0.809–4.670	0.138			
Postoperative nadir platelet count	0.989	0.987–1.000	0.041			
Preoperative hemoglobin	0.984	0.967–1.001	0.062	0.969	0.946–0.993	0.010
Postoperative nadir hemoglobin	0.972	0.947–0.998	0.036			
Intraoperative Red blood cells use	1.082	0.066–17.767	1.00			
Intraoperative fresh frozen plasma use	1.478	0.314–6.958	0.916			
Intraoperative platelets use	4.952	0.998–24.577	0.034			
Procedure time	1.007	1.000–1.013	0.052			
CPB duration	1.009	1.000–.018	0.043			
Aortic cross-clamp time	1.011	0.999–1.023	0.072			
DHCA duration per 5 mins	1.031	1.007–1.056	0.01	1.197	1.052–1.362	0.006
Lowest rectal temperature (°C)	1.022	0.680–1.534	0.918			
Lowest nasopharyngeal temperature (°C)	0.655	0.448–0.957	0.029			

*BMI* Body mass index, *CAD* Coronary artery disease; Renal insufficiency, preoperative serum creatinine > 1.2 mg/dL, *CPB* Cardiopulmonary bypass

### Short-term outcomes

The primary outcome analysis demonstrated no difference in 90-day mortality (0% v 3.6%; *p* = 0.948) between AKI patients and non-AKI patients. Table 4 compares the varying 90-day mortality among the 4 subgroups (0.0% in those without AKI, 2.6% in those with stage 1, 0.0% in those with stage 2, and 14.3% in those with stage 3). The 90-day mortality rate among stage 3 patients was significantly higher than that among non- AKI patients (*p* < 0.01).

**Table 4:** Relationship of AKI with length of stay, ventilation time and mortality. (*n* = 123)

Variables	No AKI	Stage		
		1	2	3
Hospital length of day (d)	12 (9,17.5)	12 (10,15.5)	18.5 (13.25,24) ^*^	26 (21,30) ^*^
Intensive care unit stay (d)^#^	4 (3, 6)	5 (3.75, 6)	6 (4.5, 16) ^*^	20 (9, 27) ^*^
Mechanical ventilation time(h)^#^	48 (33, 90.5)	67(46, 91)	96 (66, 253) ^*^	418 (93, 543) ^*^
90-d mortality (n)	0(0.0%)	1(2.7%)	0(0.0%)	1(14.3%)^*^

^*^P less than 0.05 compared with patients with non-AKI

^#^ P less than 0.05 compared patients with AKI and patients with non-AKI

In terms of secondary outcomes, significant differences were detected in the MVT (75[48–117] hours vs 48 [33–90.5] hours; *p* = 0.001) and LOIS (5 [4–8] days vs 4 [3–5.8] days; *p* = 0.003) between the AKI and non-AKI groups, while the p-LOHS (14 [10–20] days vs 12 [9–17.5] days; *p* = 0.057) showed no difference. However, in the sub-group analysis, patients both with AKI stage2 and stage3 AKI also had a significantly prolonged p-LOHS (18.5 [13.25–24] days vs 12 [9–17.5] days; *p* = 0.023; 26 [21–30] days vs 12 [9–17.5] days; *p* = 0.001) compared with the non-AKI group. In addition, the MVT, LOIS and p-LOHS showed no difference between the stage 1 AKI and non-AKI groups (*p* > 0.05).

## Discussion

In this observational study, we observed a high incidence of postoperative AKI (45%) after PEA. We identified that a lower preoperative platelet count, lower preoperative hemoglobin concentration and prolonged DHCA duration were independent risk factors through multivariate logistic regression analysis. The MVT, LOIS and p-LOHS increased with increasing AKI severity. There was a significant difference in 90-day mortality between patients reaching stage 3 AKI and patients without AKI.

As a result of the different diagnostic criteria for AKI and varied inclusion criteria among different previous studies, the incidence of AKI after cardiothoracic surgery under DHCA ranges from 11 to 55%, and the renal replacement therapy (RRT) rate is approximately 1.2 to 11% [25–28]. Initially, we demonstrated an incidence of 45% of postoperative AKI after PEA and reported a 3.3% rate of RRT in our study, which is similar to the results of previous studies on cardiothoracic surgery under DHCA. Due to the potentially high incidence of postoperative AKI in patients undergoing PEA, perioperative renal protective strategies should be considered with priority in clinical trials involving such patients.

This study demonstrated a significant increase in the MVT, LOIS and p-LOHS in patients with stage 3 and stage 2 AKI, with an additional significant influence of stage 3 AKI on increased 90-day mortality. However, no difference was detected between patients with non-AKI and stage 1 AKI in any of the primary outcomes. In addition, we observed no significant difference in mortality between the AKI and non-AKI groups in the present study, as a result of the relatively lower intrinsic mortality rate and insufficient sample size to detect a difference.

In this study, the logistic regression model identified that a lower preoperative platelet count, lower preoperative hemoglobin concentration and prolonged DHCA duration were independent risk factors for AKI. Previous literatures [29, 30]considered that age was one of the risk factors for AKI after cardiovascular surgery. The mechanisms of age-associated AKI might include aging of the kidneys, the influence of oxygen delivery to the kidneys, and the invasion of accompanying diseases to kidneys. However, age was not found to be an independent risk factor in this study, probably due to the relatively young age of the CTEPH patients [31] and the narrow age distribution in this study.

To achieve improved visualization, PEA is performed under CPB with DHCA, which not only provides a bloodless operative field for surgeons to perform a thorough distal-endarterectomy [32], but also provides brain protection to some degree. However, in our study, we found that a longer duration of DHCA may lead to a higher incidence of AKI, which might be explained by the complex processes of DHCA, including cooling, rewarming and hypothermic circulatory arrest.

The association of postoperative AKI with a prolonged DHCA duration has been discussed. On one hand, Mori et al. [27] reported that the duration of DHCA was a predictor of postoperative AKI. They believed that hypoxia-induced renal damage during DHCA was the underlying mechanism of AKI, which demonstrated the relationship between a prolonged duration of DHCA and an increased incidence of AKI. In addition, George et al. [33] found that short circulatory arrest time was more beneficial to patients than a long circulatory arrest duration. On the other hand, in the study by Hui Zhou et al. [18], they found that an extended CPB duration was an independent risk factor. Although DHCA was not a potential risk factor for AKI in the univariate analysis, it was closely related to a prolonged CPB duration compared with moderate hypothermic circulatory arrest (MHCA). Similarly, Englberger et al. [26] also found that DHCA was not associated with AKI. They explained this result by the protective effects of prolonged DHCA on organ function, which counterbalanced the kidney damage expected from a prolonged CPB duration. However, it is contradictory to their argument regarding the protective function of DHCA that the incidence of AKI gradually increased in their study with a DHCA duration exceeding 30 min.

Similar to previous studies [26, 27, 34–40], our study showed that a lower preoperative hemoglobin concentration was an independent risk factor for postoperative AKI after PEA. The literature on the rate of postoperative AKI in anemic patients is four-fold the rate in patients who are not anemic, which might be due to abnormal iron homeostasis [34–36, 41–43], reduced renal oxygen supply, worse oxidative stress [44], and impaired hemostasis [35]. The hemoglobin concentration determines the arterial oxygen content, which has an important role in tissue oxygen delivery. Lower hemoglobin concentrations would therefore decrease oxygen delivery to the kidneys, especially to the vulnerable renal medulla [45]. The adverse consequences of a lower hemoglobin concentration are likely enhanced further during PEA [46]. Lower hemoglobin concentrations may also improve renal oxidative stress, because red blood cells (RBCs) have important antioxidant functions [45]. Hemoglobin induces platelet activation and apoptosis in a concentration-dependent manner [47]. Lower hemoglobin concentrations activate platelets and in turn promote platelet aggregation and clot formation, which may cause damage to the renal vasculature from embolic events [48].

In the present study, we found a significant association between the preoperative platelet count and postoperative AKI, indicating that the magnitude of the decreased platelet count was significantly correlated with the severity of postoperative AKI. This finding is consistent with those from studies by Miklos D et al. [37] and Wail et al. [49], both of which observed a significant association between the nadir platelet counts and AKI. The causal relationship between postoperative AKI and the platelet counts has not yet been determined yet. We can only speculate that they might be related to the “microthrombosis”. Thrombotic microangiopathy can be manifested in many diseases, and its characteristics are thrombocytopenia, microangiopathic anemia, and organ injury, including AKI [50].

Reduction in the preoperative platelet count may be related to platelet consumption caused by microthrombosis. Therefore, a decrease in the preoperative platelet count may indicate not only macroembolism, such as pulmonary embolism and deep venous thrombosis, but also microthrombosis formed with a similar pathophysiologic mechanism in CTEPH patients before surgery. This hypothesis may account for both the severity of and susceptibility to AKI in CTEPH patients.

Since PEA is the most effective therapy for CTEPH, reducing AKI after PEA will greatly improve surgical outcomes. In this study, all independent variables associated with AKI after PEA were modifiable. If the observed association between these 3 factors— preoperative platelet count, preoperative hemoglobin concentration and duration of DHCA—and AKI after PEA is causal, then treating or avoiding these factors would reduce the incidence of AKI after PEA. There are some feasible measures with better risk-benefit profiles, such as preoperative iron and vitamin supplementation or administration of erythropoietin, cessation of drugs that affect blood clotting before surgery, minimization of hemodilution, management of antifibrinolytic drugs, and positive treatment of excessive bleeding [35]. Simultaneously, if the duration of DHCA is prolonged due to the complexity of the procedure, it is necessary to be vigilant and strictly monitor renal function.

### Study limitations

This study has several limitations. First, because this was a retrospective study, missing data and inaccurate record keeping were inevitable. The rate of AKI and the short-term outcomes of patients may have been estimated improperly in this study. As a result, only an association between the three risk factors and AKI can be demonstrated, and a cause effect relationship cannot be assumed. Furthermore, we only focused on short-term outcomes, and the long-term outcomes were not analyzed. Second, the effects of unknown or unmeasured confounders on the observed associations between the risk factors and AKI (e.g., perioperative use of nephrotoxic drugs) cannot be ruled out. Third, although we are one of the largest centers in Asia, the sample size is still small due to the low incidence of CTEPH and the late implementation of surgical techniques.

## Conclusion

This retrospective study first reported the incidence (45%) of postoperative AKI after PEA using the KDIGO criteria, and confirmed that AKI has a negative impact on short-term outcomes, including the MVT, LOIS and p-LOHS. Only stage 3 AKI was associated with a significant increase in 90-day morbidity. In the multivariate analysis, lower preoperative platelet count, lower preoperative hemoglobin concentration and prolonged DHCA duration were independent risk factors for postoperative AKI. We recommend that the preoperative platelet count, preoperative hemoglobin concentration and DHCA duration be used as predictors so that postoperative AKI after PEA can be identified earlier and treated promptly.

## Data Availability

The datasets used or analyzed during the current study are available from the corresponding author on reasonable request.

## Abbreviations

AKI: Acute kidney injury
Cis: Confidence intervals
CPB: Cardiopulmonary bypass
CTEPH: Chronic thromboembolic pulmonary hypertension
DHCA: Deep hypothermic circulatory arrest
IQR: Interquartile ranges
KDIGO: Kidney Disease Improving Global Outcomes
LOIS: Length of intensive care unit stay
MHCA: Moderate hypothermic circulatory arrest
MVT: Mechanical ventilation time
PEA: Pulmonary endarterectomy
PH: Pulmonary hypertension
p-LOHS: Postoperative length of hospital stay
RBCs: Red blood cells
ROC: Receiver operating characteristic
RRT: Renal replacement therapy
SCr: Serum creatinine
SD: Standard deviation
